# Vitamin D and Calcium as Key Potential Factors Related to Colorectal Cancer Prevention and Treatment: A Systematic Review

**DOI:** 10.3390/nu14224934

**Published:** 2022-11-21

**Authors:** Stephanie Marie Cruz-Pierard, Teresa Nestares, Francisco J. Amaro-Gahete

**Affiliations:** 1 Department of Physiology, University of Granada, 18016 Granada, Spain; 2 Centro de Investigación Biomédica (CIBM), Instituto de Nutrición y Tecnología de los Alimentos “José Mataix” (INYTA), Universidad de Granada, 18016 Granada, Spain; 3 PROmoting FITness and Health through Physical Activity Research Group (PROFITH), Department of Physical and Sports Education, Faculty of Sports Science, University of Granada, 18011 Granada, Spain; 4 Centro de Investigación Biomédica en Red Fisiopatología de la Obesidad y Nutrición (CIBERobn), Instituto de Salud Carlos III, 28029 Madrid, Spain

**Keywords:** calcium, colorectal cancer, prevention, treatment, vitamin D

## Abstract

Colorectal cancer (CRC) is currently considered one of the most common and lethal types of tumors. Nutrition is of notorious relevance, given its influence in CRC prevention and treatment. This systematic review aimed to revise and update the state of knowledge regarding the potential role of vitamin D and calcium as key factors involved in the prevention and treatment of CRC. A literature search was performed in PubMed and Web of Science. A total of eight studies were finally included in the present review. Vitamin D showed a protective role by promoting transcriptomic changes associated with antitumor effects. However, no significant effects of vitamin D were noted in the relapse-free survival of patients at 5 years. On the other hand, previous scientific evidence demonstrated that calcium regulates the expression of colonic proteins that decrease cell proliferation and increase cell differentiation. Nevertheless, an increased risk of associated serrated adenomas was found in response to calcium and calcium + vitamin D supplementation. Moreover, supplementation with both nutrients showed positive changes on relevant CRC biomarkers including TGFα, TGFβ1, APC, β-catenin and E-cadherin. In conclusion, vitamin D supplementation seems to have a protective effect in the prevention and treatment of CRC, while calcium intake showed contradictory effects as a prevention or treatment tool; therefore, further studies are necessary to well understand its relevance in patients with CRC.

## 1. Introduction

Cancer is defined as the rapid proliferation of abnormal cells that exceeds the usual limits of damage or aging, and, in certain situations, these cells invade other organs (metastasis), outnumbering healthy cells and altering their common functions [1,2]. The prevalence and incidence of cancer has increased dramatically in recent decades, making cancer the second disease with the highest mortality rate worldwide [2]. It affects both sexes depending on various risk factors such as age and physical, economic or social conditions [2].

Data provided by the World Health Organization WHO [2] show that, in 2020, the types of cancer with the highest incidence were breast cancer (2,260,000 cases and 685,000 deaths), lung cancer (2,210,000 cases and 1,800,000 deaths), colorectal cancer (CRC) (1,930,000 cases and 916,000 deaths) and prostate cancer (1,410,000 cases). Similarly, according to the Spanish Society of Medical Oncology (SEOM) [3], the most frequent cancers diagnosed in 2022 in Spain were prostate (30,884 new cases), breast (34,750), CRC (43,370) and lung (30,948) cancers, the last two being more prevalent in men than in women. Recent scientific advances in this clinical area have supposed that between 30 and 50% of cancers can be prevented through the control of specific risk factors [2].

Considering the above-mentioned data, CRC has been positioned as one of the most common and lethal types of cancer [2,3]. Its presentation is highly dependent on age and sex; men older than 50 years are the population with higher cumulated incidence [4]. Interestingly, an increase of CRC cases of 2.4% and 1% per year in adults aged 20 to 29 and 40 to 49, respectively, has been recently reported [4]. This increment has been attributed to different risk factors including (i) non-modifiable factors (e.g., family or personal history of adenomas or colon cancer, aging, etc.) and (ii) factors related to lifestyle such as toxic habits (e.g., alcohol or tobacco consumption, among others), obesity, physical inactivity, composition of the intestinal microbiota and even the intake of certain pro-inflammatory foods (e.g., red meats or sausages) [5].

Nutrition represents an aspect of great relevance, since it is able to influence both the prevention and the treatment of CRC [4,6,7,8]. It has been previously described that specific food groups can exert protective or prompting effects on CRC. Specifically, a meta-analysis by Vieira et al. [8] concluded that a high intake of red meat or sausages (i.e., over 100 g/day of meat) and alcohol (i.e., over 10 g/day of ethanol) could increase the risk of developing this type of cancer by 12 and 7%, respectively. On the other hand, they also reported that an elevated intake of dairy (i.e., over 200–400 g/day) and whole grains (i.e., over 90 g/day) showed a protective effect against CRC, reducing the risk of its development by 13 to 17%, respectively. Lastly, vegetables (i.e., 100 g/day) and fish (i.e., 100 g/day) consumption seems to be also associated with a lower risk of developing CRC [8].

Dairy products are generally avoided by a certain population due to myths, potential food allergies or intolerances, or sustainability and animal protection issues [9,10]. However, there are robust scientific evidence reporting their positive derived effects on general health [11] and, specifically, on CRC prognosis [12,13]. Low-fat milk consumption has been inversely associated with the incidence of CRC [12]. It should be noted that there is no evidence of any harmful effect of its consumption [12]. Zhang et al. [11] concluded that an increase of milk consumption to 200 mL/day was related to a lower risk of developing not only CRC (10% lower) but also several cardiometabolic pathologies such as hypertension, obesity and osteoporosis. Dairy consumption is also currently recommended during CRC treatment, since a high intake of dairy products lowers the CRC-related mortality risk [13].

Nowadays, the reasons that potentially explain why dairy products consumption is related to a lower incidence of CRC and its better prognosis are still unknown. However, one plausible explanation could be the presence of a high content of vitamin D and calcium in dairy products, both related to the pathophysiology of this cancer at the molecular level [14,15,16]. 

Vitamin D is a group of fat-soluble prohormones with important physiological functions including calcium–phosphorus homeostasis, bone formation, and proper functioning of the immune and musculoskeletal systems [15,17]. Vitamin D can be found in two forms, vitamin D2 (ergocalciferol) and vitamin D3 (cholecalciferol) [17]. In humans, most of vitamin D3 is synthesized at the cutaneous level from 7-dehydrocholesterol in the presence of sunlight [17]. However, it can also be obtained from foods such as dairy products, fish or eggs, among others [15,17]. It is important to mention that different kinds of milk, such as the skimmed one, have been enriched with vitamin D for several years. A previous study reported that the daily recommendation levels of vitamin D for the Spanish population are 15 μg and 20 μg for the populations aged 20 to 59 years and >60 years, respectively [18].

Vitamin D is involved in molecular pathways related to CRC development [15]. Important mechanisms associated with its functions play a key role in both (i) the decrease of COX-2 gene (cyclooxygenase 2) expression and (ii) the increase of 15-PDGH (enzyme catalyzing prostaglandins degradation) expression, therefore locally lowering prostaglandins levels in tumors (favoring, in a similar way, carcinogenesis by inhibiting the apoptosis of cancer cells) [15]. Vitamin D has also inhibitory functions affecting the growth of colon, breast and prostate tumor cells via the vitamin D receptor (VDR) [19]. Moreover, it has been demonstrated that vitamin D blocks β-catenin-mediated gene transcription in cultured SW480-ADH, Caco-2, and HT-29 CRC cells via inducing VDR binding to β- catenin, which subsequently reduces the formation of the TCF4/β-catenin transcriptional complex [19]. β-catenin is thus capable of activating the transcription of genes whose protein products regulate cell proliferation [19].

Calcium is an essential mineral in bone structure and also has an essential role in the transmission of nerve impulses [20]. Its recommended daily intake varies depending on the age, sex and physiological status of an individual [18]. For the Spanish population, men from 20 to 59 years and women from 20 to 49 years, the recommended daily intake is 1000 mg, while for men > 60 years and women > 50 years it is 1200 mg [18]. 

There are several molecular pathways that explain why calcium intake is considered a protective nutrient in CRC prevention. Concretely, calcium participates in cell differentiation and proliferation, as well as in intercellular connections and signal transduction cascades, influencing cell cycle regulatory genes such as p 53, K-ras and epidermal growth factor, among others, all of them factors that play a key role in the pathogenesis of CRC [21,22]. Moreover, calcium obtained by diet binds to bile acids at the intestinal level, acquiring the insoluble calcium soaps form [21,23]. It prevents the cytotoxic effects caused by fatty acids in the intestine (i.e., carcinogenic and proliferative effects), protecting the integrity of the mucous membrane [21,23].

The separation of the effects of calcium and vitamin D seems to be unfeasible, since the blood concentrations of vitamin D directly affect the calcium concentrations and vice versa, in relation to their effects on the regulation of the transport of molecules in the intestinal epithelium [14]. This interaction has been evidenced by a recent study that demonstrated that calcium supplementation reduced the recurrence of colorectal adenoma only in patients with blood levels of vitamin D (25 (OH) D) greater than 29.1 ng/mL [14].

Taking into consideration the above-mentioned scientific evidence, the present work aimed to revise and update the state of knowledge regarding the potential role of vitamin D and calcium as key factors involved in the prevention and treatment of CRC. 

## 2. Materials and Methods

### 2.1. Protocol

A systematic review of the literature was conducted strictly following the PRISMA methodology [24] and was registered in the International Prospective Register for Systematic Reviews (PROSPERO; ID: CRD42022311086). 

### 2.2. Search Strategy

Two adapted search chains with MeSH terms were developed for the PubMed and Web of Science databases (Appendix A). We restricted our review to those manuscripts published during the last 10 years, since our aim was to consider the most updated information regarding this field of knowledge.

A total of 67 and 255 articles from PubMed and Web of Science were retrieved as potential candidates to be included in the present review. The last searches were performed on 5 November 2022. 

### 2.3. Inclusion and Exclusion Criteria

The inclusion and exclusion criteria can be found in Table 1.

### 2.4. Methods to Avoid the Risk of Bias

The review protocol was conducted based on the Cochrane Manual for Systematic Reviews of Interventions [25], using its tool to assess the risk of bias of the included studies.

### 2.5. Data Extraction Method

**Figure 1:**
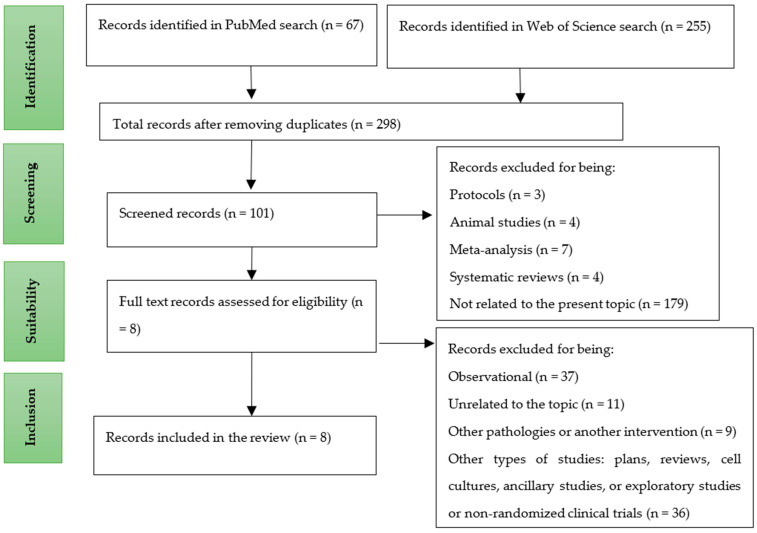
Article selection diagram.

A total of 322 works were identified after the initial search. After excluding duplicates, protocols and those articles that did not meet the inclusion criteria, 8 eligible studies were finally included (Figure 1). Specific descriptive information of each study (i.e., authors, year of publication, total number of participants, intervention, evaluation instruments, results and conclusions) is presented in Table 2. 

## 3. Results

The eight studies included in this work were double-blind randomized clinical trials [14,26,27,28,29,30,31,32]. A total of 5308 individuals were considered, all of them suffering from CRC or being at risk of its development.

Controversial findings were noted in the included studies. On the one hand, it was suggested that vitamin D supplementation did not significantly improve the relapse-free survival or overall survival at 5 years in patients with CRC [26,29,30]. However, Protiva et al. [14] established that vitamin D supplementation showed a significant increase in the expression of VDR transcriptional genes and of genes of immune and inflammatory pathways. 

Regarding calcium supplementation, Aslam et al. [28] concluded that treatment with both Aquamin ^®^ (composed of 30% calcium plus other minerals) and calcium monotherapy resulted in the upregulation of (i) many proapoptotic proteins, (ii) cytokeratins, (iii) cell-to-cell adhesion molecules and (iv) basement membrane components and in the (v) downregulation of nucleic acid proliferation and metabolism compared to placebo, together with promoting a preventive effect against CRC [28]. In contrast, Crockett et al. [27] suggested that supplementation with calcium carbonate or calcium plus vitamin D increased the risk of sessile serrated adenomas or colorectal polyps 6 to 10 years after starting with this supplementation, especially in women and smokers. 

Effects of the combined use of vitamin D and calcium supplements were also observed [30,31,32]. Baron et al. [30] determined that the supplementation of vitamin D, calcium or both did not significantly reduce the risk of recurrent colorectal adenomas over 3 to 5 years. However, Tu et al. [31] and Ahearn et al. [32] showed beneficial effects of these nutrients and their combination on important CRC biomarkers such as TGFα, TGFβ1 (increase) [31], APC (increase), β-catenin (decrease) and E-cadherin (increase) [32], concluding that calcium and vitamin D could be chemopreventive substances in CRC [32]. 

**Figure 2:**
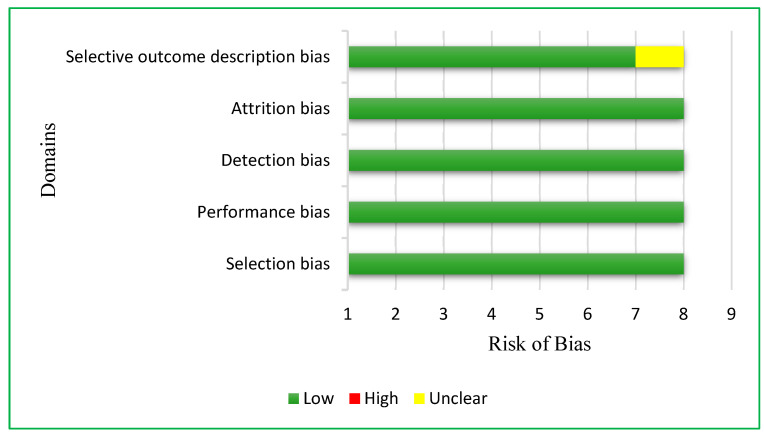
Risk of bias in the analyzed articles.

Concerning the risk of bias of the included studies (Figure 2), the one by Aslam et al. [28] showed an unclear risk of bias by describing some of the results in a selectively way, presenting only some parts of the achievements. 

## 4. Discussion

The present systematic review aimed to revise and update the state of knowledge regarding the potential role of vitamin D and calcium as key factors involved in the prevention and treatment of CRC. In general terms, we found that vitamin D is a protective factor for CRC prevention, while this micronutrient seems not to have clear effects during CRC treatment. However, controversial results have been obtained regarding calcium supplementation, a fact that questions its use in both the prevention and the treatment of CRC. 

This systematic review updates the previous evidence about the role of vitamin D and calcium on CRC prevention and treatment [21,33]. We found that vitamin D has a preventive effect on CRC at the genetic level [14], a result that concurs with those obtained by Ferrer-Mayorga et al. [34] who concluded that 1,25(OH)2 D3 protects against CRC by regulating intestinal stromal fibroblasts, suggesting that the expression of VDR and vitamin D-related genes in these cells could be the reason behind this protective effect [34].

Although no significant effects were noted in the treatment of CRC after vitamin D supplementation [26,29]—which coincides with what was observed in the study by Baron et al. [30]—other works have suggested positive effects, to a lesser extent, on survival in these patients [26,29,35,36,37]. Specifically, a systematic review and meta-analysis carried out by Vaughan-Shaw et al. [36] showed that vitamin D supplementation promoted a 30% reduction in overall adverse survival outcomes, a 24% reduction in CRC-specific death and a 33% reduction in disease progression or survival. Similarly, Zhou et al. [37] found an inverse relationship between 25(OH)D concentrations and CRC risk. Concretely, they found that patients in the highest quartile of plasma vitamin D levels had a 13% lower risk of CRC development and a 20% lower risk of overall death [37]. 

The different types of populations analyzed in each study could be of scientific and clinical importance. On the one hand, in the meta-analysis by Keum et al. [35], most of the studies included apparently healthy individuals, menopausal women, people with a recent diagnosis of cancer or patients with metabolic diseases. On the other hand, Kimmie et al. [26], Urashima et al. [29] and Baron et al. [30] only included patients with CRC or digestive advanced cancer. Similarly, in the study by Zhou et al. [37], the participants had no history of cancer at baseline. In this sense, the American Cancer Society [38] reported that a localized CRC (i.e., stages I or II, without metastasis) has a relative survival rate at 5 years of 89% to 90%, while a type IV CRC with distant metastasis registered a survival rate of 14% to 15% at 5 years.

Regarding calcium intake, the present data show an increased risk of serrated adenomas or colorectal polyps after long-term supplementation with this mineral [27]. The increased risk of polyps attributed to calcium supplementation was higher in women and smokers [27], a finding that was also obtained by García-Lopez et al. [39]. However, no reasons were provided for the explanation of these results [39]. Similarly, it was also demonstrated that smokers had a 2.5-fold increased risk of developing serrated polyps and a 13% increased risk of developing conventional adenomas compared with non-smokers [40]. Therefore, it could be plausible that tobacco consumption could interact with calcium supplementation, increasing the risk of suffering from CRC [40]. The increased risk of serrated polyps observed in smokers may be explained by specific molecular mechanisms [41], since tobacco consumption increases the risk of mutations causing malignant transformations in the colorectal mucosa through the dentate pathway [41]. Serrated polyps usually show BRAF gene mutation and, according to Bailie et al. [41], there are strong correlations between smoking and (i) microsatellite instability, (ii) a cytosine phosphoguanine (CpG) island methylator-positive phenotype and (iii) BRAF mutations. 

The above-mentioned results are contradictory to those obtained by a previous meta-analysis that indicated a protective effect of calcium supplementation against the development of adenomas [33]. Crockett et al. [27] concluded that dietary calcium intake was not associated with the development of serrated adenomas, a consistent finding also reported by Meng et al. [42]. These findings could be explained by the fact that calcium and vitamin D supplementation seems to modify the expression of β-catenin, E-cadherin and the APC/β-catenin ratio, all of them well-known factors associated with a low risk of colorectal neoplasms [32]. Furthermore, Tu et al. [31] suggested positive changes in TGFα and TGFβ1 expression in response to the above-mentioned supplementation.

This systematic review has some limitations that should be addressed. Firstly, the comparison of the examined effects across studies was hard since different studies used different supplement dosages and combinations. Furthermore, a limitation of this revision is that, due to the high heterogeneity of the included studies, it was not feasible to carry out a meta-analysis. Therefore, more randomized controlled trials with larger sample sizes, well designed and with a low risk of bias are needed [33,43].

## 5. Conclusions

In summary, according to the available evidence examined by this systematic review, supplementation with 800 IU/day of vitamin D would have a protective effect in the prevention of CRC and could modify relevant CRC biomarkers, collaborating in the treatment of this pathological condition even when it is combined with 2 g of calcium. In contrast, supplementation with 1200 mg/day of calcium seems to have a negative effect in terms of CRC prevention, especially in women and long-term smokers. More studies are therefore needed to robustly determine the effects of vitamin D and calcium supplementation on the prognosis and treatment of CRC.

## Figures and Tables

**Table 1:** Inclusion and exclusion criteria.

Inclusion Criteria	Exclusion Criteria
Patients with colon or colorectal cancer (CRC). Healthy individuals or those at risk of developing CRC Articles published from 2012 to 2022. Manuscripts in Spanish, English or French. Randomized controlled trials (RCT)	Systematic reviews, meta-analyses, observational studies, letters to the editor, short communications and other types of studies such as plans, reviews, cell cultures, ancillary studies, exploratory studies or non-randomized clinical trials Animal studies

**Table 2:** Main characteristics of the studies included in the systematic review.

Author/Year of Publication	Number of Participants	Intervention Prevention/Treatment	Evaluation Instruments	Conclusions
Kimmie et al. [26].	N = 139 participants with advanced or metastatic colorectal cancer (CRC). Age: between 47 and 65 years.	Treatment mFOLFOX6 + chemotherapy with bevacizumab every 2 weeks (A) High-dose group (n = 69): 8000 IU/d of vitamin D3 (2 capsules 4000 IU) in the first cycle and then 4000 IU/day in subsequent cycles. (B) Standard dose group (n = 70): 400 IU/day of vitamin D3 throughout the period (1 capsule of 400 IU vitamin D3 and 1 placebo during the first cycle due to blinding) Until disease progression, intolerable toxicity, or withdrawal of consent.	Progression-free survival (PFS) → log-rank test and supporting Cox proportional hazards model Objective tumor response rate (ORT), overall survival (OS) and change in plasma level of 25(OH)D. Adverse events related to supplementation	High-dose vitamin D3 supplementation vs. standard dose + chemotherapy in metastatic CRC → non-significant but clinically relevant PFS difference
Crockett et al. [27].	N = 2259 participants with at least one adenoma removed by colonoscopy. Age: between 51 and 65 years.	Prevention (A) Calcium carbonate (1200 mg of elemental calcium/day) (n = 419) (B) Vitamin D3 (1000 IU/day) (n = 420). (C) Both supplements (n = 421) (D) Placebo (n = 415) Until first planned surveillance colonoscopy at 3 or 5 years.	Block Brief 2000 Questionnaire: Demographics, Family/Medical History, Supplements/Medications, Health Habits, and Diet Height and weight: self-report or measurement → body mass index (BMI) Blood levels of 25(OH)D, calcium and creatinine Colonoscopies or other colorectal imaging Categorization of reference samples (extraction of pathology records in clinical centers) → SP subtyped as HP (MVHP, GCHP, MPHP), SSA/Ps (with or without cytological dysplasia) and TSA.	Calcium or calcium + vitamin D supplements increased risk of SSA/Ps Weigh as potential confounder in such results Dietary calcium intake was not associated with SP or SSA/P
Aslam et al. [28].	N = 30 healthy adults but with an increased risk of colon cancer based on their medical history. Age: between 18 and 80 years	Prevention (A) Aquamin ^®^ capsules (n = 10) → 800 mg of calcium/day + magnesium and other trace minerals (B) 800 mg of calcium carbonate/day (n = 10) (C) Maltodextrin (n = 10) → placebo For 90 days	Basal serum calcium levels → NIH DHQ II. Before and after intervention → colon biopsies and stool samples Quantitative histology and immunohistochemistry → expression of Ki67 (proliferation marker), CK20 and p21 (differentiation indicators) Quantitative morphometry (crypt length) Proteomic evaluation (heat map)	Aquamin ^®^ was effective at increasing proapoptotic markers, cytokeratins, cell-to-cell adhesion molecules and basement membrane components and at decreasing proliferation and metabolism of nucleic acids Calcium alone also altered expression of many proteins referred above.
Urashima et al. [29].	417 participants with clinical stages I to III digestive tract cancers (48 colorectal, 2%). Age: between 58 and 75 years.	Treatment (A) Vitamin D3: 2000 IU/d, 2 capsules per day (n = 251) (B) Placebo: 2 capsules per day (n = 166)	Relapse-free survival time (RFS) until relapse or death Overall survival time (OS) until death Computed tomography/positron emission tomography, MRI → rule out relapse of cancer Self-reported adherence interview → each visit/phone calls 25(OH) D levels → radioimmunoassay Single-nucleotide polymorphisms (SNPs) associated with the vitamin D receptor	In cancer of the digestive tract, vitamin D supplementation did not produce significant improvements of RFS after 5 years of follow-up compared to the placebo group
Protiva et al. [14].	20 healthy participants with a slightly increased risk of colorectal cancer (personal or close family history). Age: between 50 and 66 years.	Prevention Two crossover studies of 4 weeks: (A) Western-type diet (2200 calories, 20% protein, 40% fat, 400 g of calcium and low in vitamin D). After 4 weeks of washing with a normal diet, 2 g of calcium carbonate per day was added (n = 10) (B) Western-type diet + 20 IU of 1,25(OH)2 D3 (Roche) per day distributed over 2 meals. After 4 weeks of normal diet washout, 2 g of calcium carbonate was added every day for 1 month (n = 10)	Genetic expression of the complete genome in mucosal biopsies of the rectosigmoid colon Calcium concentrations in serum and urine	1,25-dihydroxyvitamin → Significant increase in VDR transcriptional target genes (effects on the colon) Calcium supplementation → little effect on colonic gene expression Vitamin D3 → gene expression of immune and inflammatory pathways, effect was almost completely eliminated with calcium supplementation
Baron et al. [30].	2259 participating patients with recent adenomas and without colorectal polyps after complete colonoscopy Age: between 45 and 75 years.	Prevention Full factorial randomization: 2 equal pills per day: (A) Placebo (n = 415) (B) 1200 mg of calcium carbonate (n = 419) (C) 1000 IU of vitamin D3 (n = 420) (D) Vitamin D3 + calcium (n = 421) Randomization of 2 groups: (E) Calcium + Placebo (n = 295) (F) Calcium + Vitamin D3 (n = 289)	Diagnosis of adenomas in follow-up colonoscopy at 3 or 5 years	Participants supplemented with vitamin D3 → mean increase in blood 25-hydroxyvitamin D (7.83 ng/mL) compared to placebo Supplementation of vitamin D3, calcium, or both → did not significantly reduce the risk of recurrent colorectal adenomas over 3 to 5 years (43% participants had one or more adenomas)
Tu et al. [31].	92 participants with good general health and at least one adenomatous colorectal polyp diagnosed in the last 36 months. Age: between 30 and 75 years.	Prevention For 6 months: (A) Placebo (n = 23) (B) 2 g of calcium carbonate divided into 2 doses per day (n = 23) (C) 800 IU of vitamin D3 divided into 2 doses per day (n = 23) (D) Calcium + Vitamin D3 (n = 23)	Normal-appearing rectal mucosal biopsy specimens. TGFα and TGFβ1 biomarkers revealed by crypt immunohistochemistry, quantitative image analysis at baseline and 6-month follow-up	Calcium and vitamin D3 supplements → non-statistically significant increase in TGFβ 1 expression, modified TGFα expression in relation to differentiation and proliferation in mucosal crypts of patients with sporadic colorectal adenoma
Ahearn et al. [32].	92 participants with good general health and at least one adenomatous colorectal polyp diagnosed in the last 36 months. Age: between 30 and 75 years.	Treatment For 6 months: (A) Placebo (n = 23) (B) 2 g of calcium carbonate divided into 2 doses per day (n = 23) (C) 800 IU of vitamin D3 divided into 2 doses per day (n = 23) (D) Calcium + Vitamin D3 (n = 23)	Normal-appearing rectal mucosal biopsy specimens APC, β-catenin and E-cadherin biomarkers revealed by crypt immunohistochemistry, quantitative image analysis at baseline and 6-month follow-up.	Calcium and vitamin D supplements → changed expression of β-catenin (decrease of 11%), E-cadherin (increase of 51%) and APC/β-catenin ratio (increase of 16%), to reduce the risk of colorectal neoplasms Calcium and vitamin D → possible chemopreventive substances in CRC APC, β-catenin and E-cadherin → potential modifiable preneoplastic risk biomarkers

mFOLFOX6: oxaliplatin; CI: confidence interval; IQR: interquartile range; cRA: adjusted risk ratios; SP: serrated polyps; HP: hyperplastic polyps; MVHP: microvesicular hyperplastic polyps; GCHP: hyperplastic polyps rich in goblet cells; MPHP: mucin-poor hyperplastic polyps, SSA/Ps: sessile serrated adenomas or polyps; TSA: traditional serrated adenoma; DHQ II: Dietary History Questionnaire II; NIH: National Institutes of Health; Ki67: proliferation marker antigen; CK20: low-molecular-weight cytokeratin indicator of differentiation; p21: cyclin-dependent kinase inhibitor 1 or differentiation indicator CDK-interacting protein 1; 25(OH)D: plasma vitamin D; SNP: single-nucleotide polymorphisms; IR: risk index; TGFα: transforming growth factor alpha, promotes cell growth. TGFβ 1: transforming growth factor beta 1, inhibits cell growth. APC: adenomatous polyposis coli.

## Data Availability

Not applicable.

## Appendix A. Search Chains

PubMed:

(vitamin D OR 25-hydroxyvitamin D OR calcidiol OR cholecalciferol OR 25OHD) OR (calcium) AND (colorectal cancer OR colorectal tumour OR colorectal carcinoma OR colorectal neoplasm OR colon cancer OR colon tumour OR colon carcinoma OR colon neoplasm OR rectum cancer OR rectum tumour OR rectum carcinoma OR rectum neoplasm OR rectal cancer OR rectal tumour OR rectal carcinoma OR rectal neoplasm)

Web of Science:

((ALL = (vitamin D OR 25-hydroxyvitamin D OR calcidiol OR cholecalciferol OR 25OHD)) OR ALL = (calcium )) AND ALL = (colorectal cancer OR colorectal tumour OR colorectal carcinoma OR colorectal neoplasm OR colon cancer OR colon tumour OR colon carcinoma OR colon neoplasm OR rectum cancer OR rectum tumour OR rectum carcinoma OR rectum neoplasm OR rectal cancer OR rectal tumour OR rectal carcinoma OR rectal neoplasm) AND ALL = (Cross-sectional studies OR Clinical trials OR randomized controlled trials)

## References

[1] American Cancer Society (ACS) (2022). What Is Cancer?. https://www.cancer.org/en/treatment/how-to-understand-your-diagnosis/what-is-cancer.html.

[2] World Health Organization (WHO) Cancer. https://www.who.int/en/news-room/fact-sheets/detail/cancer.

[3] Spanish Society of Medical Oncology (SEOM) Cancer Figures in Spain. https://seom.org/prensa/el-cancer-en-cifras.

[4] Song M., Chan A.T., Sun J. (2020). Influence of Gut Microbiome, Diet, and Environment on Colorectal Cancer risk. Gastroenterology.

[5] Zhang L., Zou H., Zhao Y., Hu C., Atanda A., Qin X., Jia P., Jiang Y., Qi Z. (2019). Association between blood circulating vitamin D and colorectal cancer risk in Asian countries: A systematic review and dose-response meta-analysis. BMJ Open.

[6] Short V., Atkinson C., Ness A., Thomas S., Burden S., Sutton E. (2016). Patient experiences with perioperative nutrition within an Enhanced Recovery After Surgery program. surgery for colorectal surgery: A qualitative study. Colorectal Disease: Official Publication of the Coloproctological Association of Great Britain and Ireland. Color. Dis..

[7] Farinetti A., Zurlo V., Manenti A., Coppi F., Mattioli A.V. (2017). Mediterranean diet and colorectal cancer: A systematic review. Nutrition.

[8] Vieira A.R., Abar L., Chan D.S.M., Vingeliene S., Polemiti E., Stevens C., Greenwood D., Norat T. (2017). Foods and beverages and colorectal cancer risk: A systematic review and meta-analysis of cohort studies, an update of the evidence of the WCRF-AICR Continuous Update Project. Ann. Oncol..

[9] HealthyChildren.org. The Most Common Food Allergies. https://www.healthychildren.org/Spanish/healthy-living/nutrition/Paginas/Common-Food-Allergies.aspx.

[10] Tomas Pascual Sanz Institute Ten Myths about Milk and the Reality Behind It. https://www.institutotomaspascualsanz.com/diez-mitos-sobre-la-leche-y-la-realidad-que-hay-detras/.

[11] Zhang X., Chen X., Xu Y., Yang J., Du L., Li K., Zhou Y. (2021). Milk consumption and multiple health outcomes: Umbrella review of systematic reviews and meta-analyses in humans. Nutr. Metab..

[12] Barrubés L., Babio N., Becerra-Tomás N., Rosique-Esteban N., Salas-Salvadó J. (2019). Association Between Dairy Product Consumption and Colorectal Cancer Risk in Adults: A Systematic Review and Meta-Analysis of Epidemiologic Studies. Adv. Nutr. Int. Rev. J..

[13] Jin S., Kim Y., Je Y. (2020). Dairy Consumption and Risks of Colorectal Cancer Incidence and Mortality: A Meta-analysis of Prospective Cohort Studies. Cancer Epidemiol. Biomark. Prev..

[14] Protiva P., Pendyala S., Nelson C., Augenlicht L.H., Lipkin M., Holt P.R. (2016). Calcium and 1,25-dihydroxyvitamin D _3_ modulate genes of immune and inflammatory pathways in the human colon: A human crossover trial. Am. J. Clin. Nutr..

[15] Calmarza P., París A.S., López C.P., Barrio M.L., Carceller D.B. (2018). Vitamin D levels in newly diagnosed cancer patients. Hosp. Nutr..

[16] Maalmi H., Walter V., Jansen L., Boakye D., Schöttker B., Hoffmeister M., Brenner H. (2018). Association between Blood 25-Hydroxyvitamin D Levels and Survival in Colorectal Cancer Patients: An Updated Systematic Review and Meta-Analysis. Nutrients.

[17] Valero M., Hawkins F. (2007). Metabolism, endogenous and exogenous sources of vitamin D. Span. J. Bone Metab. Dis..

[18] Moreiras O., Carbajal A., Cabrera L., Cuadrado C. (2018). Food Composition Tables.

[19] Fleet J.C., Desmet M., Johnson R., Li Y. (2011). Vitamin D and cancer: A review of molecular mechanisms. Biochem. J..

[20] National Institutes of Health (NIH) Calcium. https://ods.od.nih.gov/factsheets/Calcium-DatosEnEspanol/.

[21] Bonovas S., Fiorino G., Lytras T., Malesci A., Danese S. (2016). Calcium supplementation for the prevention of colorectal adenomas: A systematic review and meta-analysis of randomized controlled trials. World J. Gastroenterol..

[22] Al-Ghafari A.B., Balamash K.S., Al Doghaither H.A. (2019). Relationship between Serum Vitamin D and Calcium Levels and Vitamin D Receptor Gene Polymorphisms in Colorectal Cancer. BioMed Res. Int..

[23] Fuszek P., Lakatos P., Tabak A., Papp J., Nagy Z., Takacs I., Horvath H.C., Lakatos P.L., Speer G. (2004). Relationship between serum calcium and CA 19-9 levels in colorectal cancer. World J. Gastroenterol..

[24] Moher D., Liberati A., Tetzlaff J., Altman D.G. (2009). Preferred reporting items for systematic reviews and meta-analyses: The PRISMA statement. PLoS Med..

[25] Higgins J.P.T., Thomas J., Chandler J., Cumpston M., Li T., Page M.J., Welch V.A. Cochrane Handbook for Systematic Reviews of Interventions Version 6.2. Cochrane. www.training.cochrane.org/handbook.

[26] Kimmie N.G., Nimeiri H.S., McCleary N.J., Abrams T.A., Yurgelun M.B., Cleary J.M., Rubinson D.A., Schrag D., Miksad R., Bullock A.J. (2019). Effect of High-Dose vs Standard-Dose Vitamin D3 Supplementation on Progression-Free Survival Among Patients With Advanced or Metastatic Colorectal Cancer: The SUNSHINE Randomized Clinical Trial. JAMA.

[27] Crockett S.D., Barry E.L., Mott L.A., Ahnen D.J., Robertson D.J., Anderson J.C., Wallace K., Burke C.A., Bresalier R.S., Figueiredo J.C. (2019). Calcium and vitamin D supplementation and increased risk of serrated polyps: Results from a randomised clinical trial. Gut.

[28] Aslam M.N., McClintock S.D., Jawad-Makki M., Knuver K., Ahmad H.M., Basrur V., Bergin I.L., Zick S.M., Sen A., Turgeon D.K. (2021). A Multi-Mineral Intervention to Modulate Colonic Mucosal Protein Profile: Results from a 90-Day Trial in Human Subjects. Nutrients.

[29] Urashima M., Ohdaira H., Akutsu T., Okada S., Yoshida M., Kitajima M., Suzuki Y. (2019). Effect of Vitamin D Supplementation on Relapse-Free Survival Among Patients With Digestive Tract Cancers. JAMA.

[30] Baron J.A., Barry E.L., Mott L.A., Rees J.R., Sandler R.S., Snover D.C., Bostick R.M., Ivanova A., Cole B.F., Ahnen D.J. (2015). A Trial of Calcium and Vitamin D for the Prevention of Colorectal Adenomas. N. Engl. J. Med..

[31] Tu H., Flanders W.D., Ahearn T.U., Daniel C.R., Gonzalez-Feliciano A.G., Long Q., Rutherford R.E., Bostick R.M. (2015). Effects of calcium and vitamin D3on transforming growth factors in rectal mucosa of sporadic colorectal adenoma patients: A randomized controlled trial: Calcium/Vitamin D and Human Gut Growth Factors. Mol. Carcinog..

[32] Ahearn T.U., Shaukat A., Flanders W.D., Rutherford R.E., Bostick R.M. (2012). A Randomized Clinical Trial of the Effects of Supplemental Calcium and Vitamin D3 on the APC/β-Catenin Pathway in the Normal Mucosa of Colorectal Adenoma Patients. Cancer Prev. Res. (Philadelphia Pa.).

[33] Veettil S.K., Ching S.M., Lim K.G., Saokaew S., Phisalprapa P., Chaiyakunapruk N. (2017). Effects of calcium on the incidence of recurrent colorectal adenomas. Medicine.

[34] Ferrer-Mayorga G., Gómez-López G.G., Barbáchano A., Fernández-Barral A., Peña C., Pisano D.G., Cantero R., Rojo F., Muñoz A., Larriba M.J. (2017). Vitamin D receptor expression and associated gene signature in tumour stromal fibroblasts predict clinical outcome in colorectal cancer. Gut.

[35] Keum N., Lee D.H., Greenwood D.C., Manson J.E., Giovannucci E. (2019). Vitamin D supplementation and total cancer incidence and mortality: A meta-analysis of randomized controlled trials. Ann. Oncol..

[36] Vaughan-Shaw P.G., Buijs L.F., Blackmur J.P., Theodoratou E., Zgaga L., Din F.V.N., Farrington S.M., Dunlop M.G. (2020). The effect of vitamin D supplementation on survival in patients with colorectal cancer: Systematic review and meta-analysis of randomised controlled trials. Br. J. Cancer.

[37] Zhou J., Ge X., Fan X., Wang J., Miao L., Hang D. (2021). Associations of vitamin D status with colorectal cancer risk and survival. Int. J. Cancer.

[38] American Cancer Society (ACS) What are the Survival Rates for Colorectal Cancer by Stage? 2022. Cancer.org. https://www.cancer.org/es/cancer/cancer-de-colon-o-recto/deteccion-diagnostico-clasificacion-por-etapas/tasas-de-supervivencia.html.

[39] García-López I.S., Gálvez-Castillejos P., Molina-Villena A.A., Swain-Saint Martin J.A., Zárate-Osorno J.I. (2019). Incidence of colorectal cancer in serrated polyps. Endoscopy.

[40] He X., Wu K., Ogino S., Giovannucci E.L., Chan A.T., Song M. (2018). Association Between Risk Factors for Colorectal Cancer and Risk of Serrated Polyps and Conventional Adenomas. Gastroenterology.

[41] Bailie L., Loughrey M.B., Coleman H.G. (2017). Lifestyle Risk Factors for Serrated Colorectal Polyps: A Systematic Review and Meta-analysis. Gastroenterology.

[42] Meng Y., Sun J., Yu J., Wang C., Su J. (2019). Dietary Intakes of Calcium, Iron, Magnesium, and Potassium Elements and the Risk of Colorectal Cancer: A Meta-Analysis. Biol. Trace Element Res..

[43] García-Morales N., Satorres C., Bustamante-Balén M. (2018). Calcium and vitamin D in the serrated neoplastic pathway: Friends or foes?. World J. Gastrointest. Pathophysiol..

